# Polysaccharide-Based Approaches for Inflammation Treatment: Anti-Inflammatory Mechanisms, Delivery Approaches and Translational Prospects

**DOI:** 10.3390/biom16071051

**Published:** 2026-07-18

**Authors:** Xiaoyu Ren, Yuan Zhou, Fei Liu, Lili Jiang, Ai Yuan, Zhouchunxiao Du, Dezhi Sui

**Affiliations:** 1 Institute of Molecular Medicine, School of Medicine, Liaodong University, Linjiang Campus, No. 116 Linjiang Back Street, Zhen’an District, Dandong 118001, China; 2 College of Pharmacy, Shenyang Pharmaceutical University, No. 103, Wenhua Road, Shenyang 110016, China

**Keywords:** polysaccharide, inflammatory diseases, anti-inflammatory mechanism, drug delivery

## Abstract

Inflammation is a physiological response of the body to infection and tissue damage; dysregulated inflammatory signaling can cause the initiation and aggravation of a variety of chronic diseases. Although anti-inflammatory drugs have been commercialized and showed beneficial effects clinically, their long-term application is often limited by loss of effects and adverse side effects. Natural polysaccharides are multifunctional biomacromolecules with strong potentials in inflammation therapy due to their inherent biocompatibility, wide immunomodulation property and drug capacity. In this Review, we offer a holistic summary of recent advances in polysaccharide anti-inflammatory strategies by coupling the characteristics of structure, biological mechanism and polysaccharide-based delivery systems. This review gives a comprehensive overview of the rational design of anti-inflammatory strategies based on polysaccharides, and also points out the current limitations of polysaccharide drugs in applications, translation difficulties and future trends.

## 1. Introduction

Inflammation is an essential biological response that protects the host against potentially injurious stimuli (including infection and tissue injury) by killing the offending agent, limiting tissue damage and initiating tissue repair [1,2]. Under physiological conditions, inflammatory responses are tightly controlled and quickly resolved when their protective roles have been fulfilled, and maintain tissue homeostasis [3]. Failure of inflammation to resolve leads to sustained immune activation, disruption of tissue homeostasis and chronic inflammatory diseases, including rheumatoid arthritis, inflammatory bowel disease and inflammatory neurodegenerative disorders [4]. As a result, chronic inflammation is the leading cause of disease burden worldwide and poses a major public health challenge.

Current pharmacological management of inflammatory diseases are mainly based on nonsteroidal anti-inflammatory drugs (NSAIDs), corticosteroids and biologic agents [5]. Although these agents have greatly improved the current treatment outcomes, their long-term clinical use remains restricted by several important issues. Prolonged treatment with NSAIDs and corticosteroids is accompanied with adverse events, such as gastric injury, osteoporosis, and metabolic disorders [6]. Biologic therapies are potent in selected patients but are expensive and associated with enhanced risk for opportunistic infections [7]. Additionally, a number of patients have an unsatisfactory response or the developed poor therapeutic response gradually during long-term treatment [8]. This has identified the advantages of safer and more efficient therapies for long-term treatment of chronic inflammatory diseases.

In recent years, naturally derived bioactive compounds such as polyphenols, peptides and polysaccharides, attract attention as promising anti-inflammatory agents because of their good biocompatibility, diverse bioactivities and multifunctionality therapeutic property [9,10]. Among them, natural polysaccharides attract more interests as they are characterized by impressive diversity, broad immunomodulation and have great potential for biomaterial synthesis [11]. Polysaccharides are long polymer molecules containing repeating monosaccharide units bonded to each other by glycosidic bonds and have great diversity in terms of their molecular weight, kinds of monosaccharides, branching, glycosidic linkages, and modification of functional groups. All of these characteristics define the properties of polysaccharides as well as their bioprofiling and therapeutic potentials. Increasing reports on the mechanism involved in modulating inflammatory responses by polysaccharides include the immune-cell remodelling, the remodelling of cytokine system, regulation of intracellular signal pathways and regulation of inflammatory environment [12,13,14]. For example, hyaluronic acid (HA) has been reported to bind to receptors, such as CD44 and some other immune-related receptors, to affect the polarization of macrophages, cytokine production, oxidative stress and tissue regeneration [15]. Besides their intrinsic activity, polysaccharides have also been extensively engineered into various advanced biomaterial platforms such as nanoparticles, hydrogels, microspheres, other stimulating delivery systems and other advanced systems, which advances their medicinal applications [16,17,18].

Herein, we provide a comprehensive and critical review of polysaccharide-based strategies for treatment of inflammatory diseases that integrates advances in polysaccharide chemistry, immunology, biomaterials and translational medicine. First, we introduce the principal families of naturally occurring polysaccharides and pay attention to their structural features and biological properties. We then review the current knowledge of structure–activity relationships and mechanisms of action of polysaccharide anti-inflammatory effects in a critical manner. Next, we summarize representative treatments and polysaccharide delivery systems for rheumatoid arthritis, inflammatory bowel disease and neurological disorders and differentiate the intrinsic biological activities of polysaccharide from their use as drug-delivery materials. Finally, the present status of challenges such as structural diversity, mechanistic studies, pharmacokinetic characteristics, standardization and clinical translation are critically assessed and prospects for future work are highlighted. Overall, the review provides a comprehensive picture of the relevant principles underlying the rational design and clinical translation of polysaccharide-based anti-inflammatory therapeutic systems.

This article was presented as a narrative review to give a comprehensive picture on recent progresses of polysaccharide-based strategies for treatment of inflammation. Relevant information was mostly retrieved from PubMed and Web of Science based on combinations of the following keywords: “polysaccharide”, “inflammation”, “anti-inflammatory”, “drug delivery”, “immune regulation”, “hyaluronic acid”, “chitosan”, “alginate”, “fucoidan”, “β-glucan” and other related words. More emphasis was put on the studies published between 2020 and 2025 to reflect the early fast-developing field, while some meaningful literature earlier than these years were included where necessary to provide background and foundation knowledge for readers. Reviews of both mechanism and translational studies were taken into consideration, and representative articles were chosen according to scientific quality, novelty and relevance to the scope of the review.

## 2. Sources and Classification

Polysaccharides are high-molecular-weight biopolymers and are built up from monosaccharide units through glycosidic bonds [19]. The amazing structural diversity originates from the difference of monosaccharide type, polysaccharide molecular weight, glycosidic linkage types, branching configuration, charges and chemical treatments [20]. These structural features not only determine the physicochemical properties of polysaccharides but also largely affect their receptor recognition and biological activities, and therapeutic potentials. Therefore, polysaccharides present various immunoregulation and anti-inflammatory properties and have attracted increasing interest for inflammation treatment as multifunctional biomaterials. Polysaccharides, according to their biological sources, can be specifically sorted into animal-, plant-, algae-, microbial- and fungal-derived polysaccharides. For ease of comparison of representative polysaccharides, sources, structural features, biological effects and medical properties of them are summarized in Table 1.

### 2.1. Animal-Derived Polysaccharides

Hyaluronic acid (HA) is a non-sulfated glycosaminoglycan composed of repeating disaccharide units of D-glucuronic acid and N-acetyl-D-glucosamine linked alternatively by β-1,4 and β-1,3 glycosidic bonds [21]. As one of the major components of extracellular matrix, HA plays important roles in tissue hydration, lubrication and healing due to its excellent water-retaining capacity and viscoelasticity [22]. The molecular weight classification of HA is inconsistent. Generally, the molecular weights of HA > 500–1000 kDa are deemed as high-molecular-weight HA [23]. However, the biological activity of HA is highly dependent on its molecular weight. High-molecular-weight HA usually has anti-inflammatory and immunosuppressive effects, promoting tissue homeostasis and maintaining extracellular matrix integrity [24]. Conversely, low-molecular-weight HA and fragmented HA are often effector molecules participating in early inflammatory responses as endogenous DAMPs. These fragments are mainly derived from hyaluronidase or reactive oxygen species fragments in tissue injury and inflammatory responses. Depending on fragment size, receptor context and local tissue environment, they stimulate angiogenesis, inflammatory signaling, immune-cell activation and extracellular matrix remodeling, respectively. Accumulation of fragments is a common phenomenon during early and post-acute tissue repairs, during which fragments facilitate tissue repair, while modulating inflammatory responses. Therefore, it is important to attribute the biological action of HA by looking both at its molecular weight and tissue physiological environment in which HA is generated.

**Table 1:** Representative polysaccharides: sources, structural characteristics, mechanisms, and therapeutic relevance in inflammatory diseases.

Polysaccharide	Source	Structural Features	Mechanisms of Action	Applications	Reference
Hyaluronic acid	Animal-derived	Linear glycosaminoglycan; repeating disaccharides	anti-inflammatory, immunosuppressive, angiogenesis	Tissue repair, osteoarthritis, wound healing	[21,22,23]
Chondroitin sulfate	Animal-derived	Sulfated glycosaminoglycan; variable sulfation patterns	Interacts with proteins and receptors; modulates inflammation and immune responses	Osteoarthritis, cartilage repair, inflammation-related disorders	[25]
Chitosan	Animal-derived	Cationic polysaccharide; β-1,4-linked glucosamine units	Antibacterial, immunomodulatory; promotes macrophage activation and tissue regeneration	Wound healing, drug delivery, tissue engineering	[26]
Pectin	Plant-derived	α-1,4-linked galacturonic acid backbone; branched domains	Modulates gut microbiota; produces SCFAs; suppresses intestinal inflammation	Gut inflammation, intestinal barrier protection	[27]
Astragalus polysaccharides	Plant-derived	Heteropolysaccharide; variable composition	Regulates cytokines, macrophage activation, oxidative stress pathways	Immune regulation, chronic inflammation	[28]
Konjac glucomannan	Plant-derived	β-1,4-linked mannose and glucose; high MW	Modulates metabolism, immune response, antioxidant activity	Metabolic disorders, inflammation, gut health	[29]
Alginate	Algal-derived	Linear copolymer (M/G blocks)	Supports drug delivery and tissue scaffolding	Drug delivery, wound healing	[30]
Fucoidan	Algal-derived	Sulfated fucose-rich polysaccharide; high heterogeneity	Interacts with immune receptors; anti-inflammatory and immunomodulatory	Cancer, inflammation, immune disorders	[31]
Xanthan gum	Microbial-derived	Branched polysaccharide; high viscosity	Forms viscous systems; stabilizes formulations; indirect biological effects	Drug formulation, controlled release systems	[32]
Sanghuang polysaccharides	Fungal-derived	β-glucans with branching; heterogeneous composition	Regulates cytokines (TNF-α, IL-6, IL-1β); enhances anti-inflammatory response	Chronic inflammation, immune regulation	[33,34]

Chondroitin sulfate (CS) is a naturally sulfated glycosaminoglycan present in nature and consists of repeating disaccharide units of N-acetylgalactosamine and D-glucuronic acid [25]. CS usually has a molecular weight of 50–100 kDa, and this varies with the biological source [35]. On the basis of the degree of sulfation and disaccharide structures, CS can be divided into several subtypes, which include CS-O, CS-A, CS-C, CS-D and CS-E [25]. The degree of sulfation, molecular weight and disaccharide structure of CS determine CS activity, and thus their interactions with extracellular proteins, growth factors and immune receptors involved in inflammation, tissue repair, immune regulation and other functions [36]. Marine CS has usually higher degree of sulfation and often possesses enhanced activities for anti-inflammatory and immune-regulation purposes [37].

Chitosan is a cationic polysaccharide obtained by partial deacetylation of chitin, which is mainly purified from crustaceans’ exoskeletons [26]. It is composed of repeating units of D-glucosamine and N-acetyl-D-glucosamine connected by β-1,4 glycosidic linkage. Due to its cationic nature and abundant primary amino groups, chitosan is biodegradable, antibacterial, mucolytic, immunomodulating. It can promote tissue regeneration and regulation of the local immune by improving cell adhesion and proliferation, accelerating the healing process of wounds and activating macrophages [26]. Furthermore, its primary amino groups provide the material pH responsiveness to the solubility, strong electrostatic interaction with negative charged biomolecule and great sites for chemical modifications, for which chitosan is one of the most versatile polysaccharide materials for biomedical applications [38].

### 2.2. Plant-Derived Polysaccharides

A major component of plant cell walls, pectin has attracted a lot of attention due to its heterogeneous immunomodulating functions [27]. Pectin consists of a homogalacturonan backbone containing D-galacturonic acid from α-1,4-linked residues interspersed with highly branched domains from rhamnogalacturonan I and II types [39]. After oral administration, pectin interacts with intestinal epithelial cells to inhibit the inflammatory signalling pathways to promote epithelial integrity and proper mucosal immune function [40]. Additionally, pectin can be easily fermented by the intestinal microflora to produce short chain fatty acids including acetate, propionate and butyrate, among others, that are important for regulating intestinal immune homeostasis and integrity [41]. These microbiota-mediated effects make pectin particularly suited for the treatment of intestinal inflammatory disorders.

Astragalus polysaccharides (APSs), that is, astragalus polysaccharides, are bioactive heteropolysaccharides isolated from Astragalus membranaceus, which is one of the most extensively used medicinal plants in traditional Chinese medicine [28]. APSs mainly comprise glucose, arabinose, galactose, rhamnose and uronic acids, the monosaccharide constituents, molecular weight and glycosidic linkage of which vary greatly depending on the plant origin, the extraction process and purification method [42]. Some studies show that APSs can affect the macrophage activity, modulate cytokines, relieve oxidative stress and regulate several signalling pathways associated with inflammation and immune homeostasis [43,44]. However, their structural variability hamper mechanistic studies and standardization.

Konjac glucomannan (KGM) is a natural water-soluble polysaccharide extracted from the tubers of Amorphophallus species [29]. KGM usually has relatively high molecular weights in the range of several hundred kDa to more than 2000 kDa [45]. In addition to its high viscosity, good water solubility, strong swelling capacity and great water-retention properties, KGM regulates the glucose and lipid metabolism by slowing down gastric emptying, attenuating glucose absorption, enhancing the sensitivity to insulin and suppressing cholesterol synthesis [46], KGM also benefits intestinal barrier function and immunomodulation, and also plays a role in resolving chronic inflammation [47].

### 2.3. Algal-Derived Polysaccharides

Alginate is an anionic polysaccharide mainly isolated from brown algae, and it has been widely studied due to its excellent biocompatibility, mild gelation characteristics and cheapness of production [30]. Alginate is made up of β-1,4 linked D-mannuronic acid (M) and α-1,4 linked L-guluronic acid (G) residues and sequentially forms the M-blocks, G-blocks and alternating MG-blocks [48]. The relative proportion and distribution of such blocks dictates the physicochemical properties and biological functions of alginate [49]. Most important, G-rich areas have the ability to bind with divalent cations, such as Ca^2+^, so the alginate can quickly form an ionic crosslink by the well-known ‘egg-box’ structure [50]. Alginate with these properties is one of the most commonly applied polysaccharides for preparing hydrogels, for drug delivery and tissue engineering.

Fucoidan is a family of sulfated polysaccharides most of which has been isolated from brown algae, having molecular weights over several hundred kilodaltons [31]. Fucoidan mainly contains L-fucopyranose units linked to each other by α-1,4 glycosidic bonds or alternatively in the α-1,3/α-1,4 pattern, the sulfate groups being mostly substituted to the C-2 and C-4 positions [51]. The molecular weight, sulfation pattern and monosaccharide constituents account for the dramatic structural heterogeneity of fucoidan, that is largely responsible for its biological activities. The high content of the negative sulfate groups allows it to interact with immune receptor, growth factors, adhesion molecules and extracellular proteins, therefore eliciting the anti-inflammatory and immunomodulatory properties of fucoidan [52].

### 2.4. Microbial/Fungal-Derived Polysaccharides

Xanthan gum (XG) is an extracellular polysaccharide produced by Xanthomonas campestris, a product of aerobic fermentation, and has been widely used in formulations in the pharmaceutical industry since it has been FDA (US Food and Drug Administration)-approved for a food additive [32]. XG is a β-1,4-linked polymer of D-glucopyranose chains that have trisaccharide side chain residues containing a mannose–glucuronic acid–mannose residue attached to the glucopyranose backbone via α-1,3 links [53]. Owing to the presence of glucuronic acid in the XG molecule and ionizable substitution groups, it can form strong hydrogen bonding and electrostatic interactions in aqueous solutions, and XG can generate high viscosity with very low concentration [54]. XG is mainly applied as an excipient in pharmaceutical formulations and as the drug matrix rather than an active anti-inflammatory substance.

Sanguang polysaccharides are major active ingredients in Phellinus linteus, which have been used in Chinese medical practice to treat chronic inflammatory diseases [33,34]. Sanguang polysaccharides are mainly composed of glucose linked by β-1,3 with β-1,6 branch point, which is structurally close to a potent immunomodulatory structure [55]. Sanguang polysaccharides can extinguish over inflammatory responses by decreasing production of inflammatory cytokines (TNF-α, IL-6 and IL-1β), and meanwhile increasing the production of anti-inflammatory cytokines [56]. Chemical modification could further improve their solubility, stability and biocompatibility and applications of these polysaccharides can also be broadened as multifunctional biomaterials for inflammation therapy [57].

Polysaccharides, although frequently seen as a group of biomacromolecules collectively, are highly diverse in monosaccharide compositions, molecular masses, glycosidic structures, branching patterns, charges, chemical modifications. Such structural variations determine their physicochemical properties, receptor recognition profiles, degradation characteristics and, most likely, their biological activities. For example, hyaluronic acid engages mostly in CD44 and RHAMM, and β-glucans are largely recognized by Dectin-1 but fucoidan is specifically recognized by selectins and scavenger receptors as a result of the high sulfation level of fucoidan, etc. As a consequence, different polysaccharides regulate inflammation through different molecular mechanisms and play different therapeutic functions in body, ranging from intrinsic immunomodulators to potent drug-carrier materials. Thus, their anti-inflammatory properties and therapeutic applications should be understood in their own unique structural characteristics and not translated across the whole group of polysaccharides.

## 3. Structure and Anti-Inflammatory Activity Relationships of Polysaccharides

The anti-inflammatory activities of polysaccharides depend on the combination of structural features rather than depending on any particular single parameter. Factors that can play a role include molecular weight, monosaccharide content, glycosidic linkages, degree of branching, higher conformation and chemical modification of polysaccharides [58]. These structural features each contribute to the physicochemical properties of polysaccharides, their binding properties for immune receptors and to the downstream signalling effects they affect. Therefore, knowledge of structure–activity relationships is important for understanding polysaccharide-mediated immunomodulation and in guiding the rational design of polysaccharides as anti-inflammatory drugs.

### 3.1. Primary Structural Determinants

#### 3.1.1. Molecular Weight

**Figure 1:**
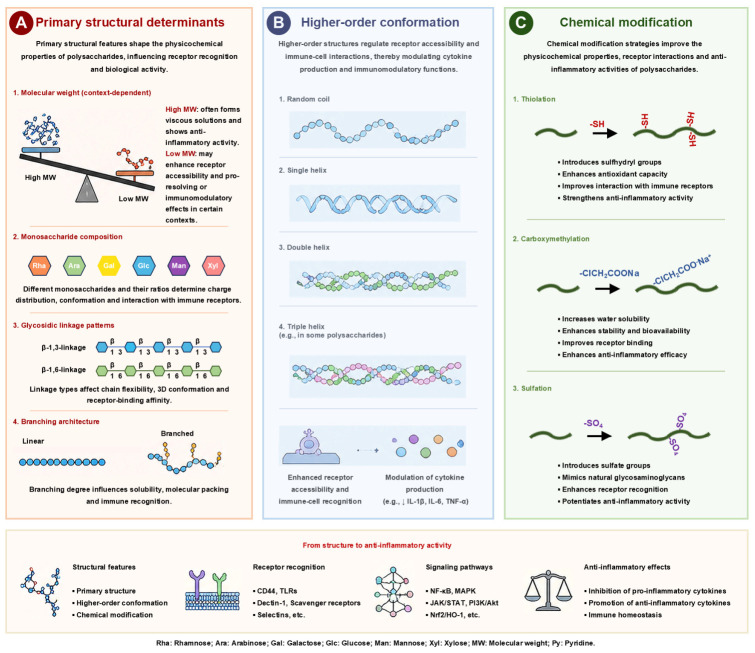
Structure-activity relationships in the anti-inflammatory activities of polysaccharides. (**A**) Primary structural moieties, molecular weight, monosaccharide composition, glycosidic linkage and branching structures, affect receptor recognition and downstream biological activities. (**B**) Global conformation regulates receptor–polymer recognition, immunological interactions and immune cell binding, which contributes to cytokine production and immunological modulation. (**C**) Chemical modification strategies that improve polysaccharides’ physicochemical properties, receptor binding and anti-inflammatory functions, such as thiolation, carboxymethylation and sulfation, are summarized. Figure is original; no copyright permission is required.

Another structural parameter that has been extensively studied is the molecular weight (weight) of the polysaccharide [59], which affects the anti-inflammatory activity of polysaccharides. In many instances, low-molecular-weight polysaccharides have better biological activity than higher-molecular-weight polysaccharides, due to better aqueous solubility, better penetration into tissues and accessibility to immune receptors [60]. For example, among several *Lycium barbarum* polysaccharide fractions, the 34.6 kDa fraction has the strongest inhibitory effect on lipopolysaccharide (LPS)-induced nitric oxide production in macrophage [61]. Molecular weight can also affect the bioavailability of polysaccharides, but it can also modify higher-order conformation and affect biological activity (Figure 1A). For example, in fucoidan, molecular weight shows a non-linear effect on gastroprotective activity, with higher-molecular-weight fractions forming random coil conformation and acting only as physical barriers, whereas intermediate-molecular-weight fractions form tighter conformations, permitting receptor-mediated actions [62].

Nevertheless, a molecular weight–anti-inflammatory activity correlation is not universal. Although lower-molecular-weight polysaccharides are often more accessible by their receptors, higher-molecular-weight polysaccharides may possess better biological activity because of better tissue retention, increased conformational stability or biophysical properties. These apparently opposing conclusions can be explained by differences in the polysaccharide sources, extraction processes, structure, disease model and assay method. Therefore, molecular weight should be considered one of the interdependent factors contributing to biological activity and not as an independent determinant of biological activity.

#### 3.1.2. Monosaccharide Composition and Proportion

Monosaccharide composition is another important factor of the polysaccharide bioactivity [63]. Different monosaccharide residues give rise to different physicochemical properties and receptor-binding profiles, leading to subsequent immune response effect. For instance, β-glucans are mostly made up of glucose residues and are well-known for their strong immunomodulation and anti-inflammatory activities [64]. Even polysaccharides with the same monosaccharide composition might have totally different biological effects because of the variations of monosaccharide ratios (Figure 1B). Comparative analyses of polysaccharides from *Dioscorea polystachya*, Rubus idaeus and Astragalus membranaceus found that the differing relative ratios of rhamnose, arabinose, xylose, mannose and glucose could lead to different suppressive effects on inflammatory mediators [65]. Hence, both monosaccharide composition and relative proportion should be considered when exploring the structure–activity relationship.

#### 3.1.3. Glycosidic Linkages and Branching Patterns

Like the type and position of glycosidic linkages, they are both crucial factors of the bioactivity of polysaccharides [66]. Among these, the β-1,3 and β-1,6 glycosidic linkages are generally linked with good immunomodulatory and anti-inflammatory activities (Figure 1C) [67,68]. Polysaccharides with mixed β-1,3 and 1,6 linkages have been reported to regulate the TLR4/MyD88/NF-κB signalling pathway and revitalize the Th17/Treg immune balance [69]. In addition, the branching architecture has an influence on biological activity on polysaccharide water solubility, molecular flexibility and three-dimensional shape. A highly branched polysaccharide is more likely to take a spatial conformation suitable for receptor recognition and immune regulation [70]. Individual linkage types and branching pattern also give influences on the biological activities, and their importance is dependent very much on the overall polysaccharide structure, and hence, have to be understood in other context of characteristics.

### 3.2. Advanced Structural Features

Beyond the primary structure, higher-order conformation is also important in the anti-inflammatory activities of polysaccharides [71]. Depending on the molecular weight, monosaccharide composition, glycosidic linkage patterns, polysaccharide chains exist in either random-coil, single-helical, double-helical, and triple-helical conformation in solution [72]. Among them, triple-helical conformations are the most conducive to being recognized by a receptor and modulate the immune system. And some polysaccharides in stable triple-helical conformation show stronger inhibition of the production of inflammatory cytokines, together with higher immune-regulating activity [73]. The relationship between higher-order conformation and biological activities is still incompletely understood because conformational changes are often accompanied by changes in molecular weight and other structural features at the same time.

### 3.3. Structural Modification Strategies

Chemical modification is a successful strategy to improve the anti-inflammatory properties of polysaccharides [74,75]. Some common chemical modification means include thiolation, carboxymethylation and sulfation, all of which positively affect the biological properties of polysaccharides in different ways. Thiolated hyaluronic acid improves the holding time of hyaluronic acid in the inflamed tissues and displays higher anti-inflammatory biological activity in experimental models of inflammatory bowel disease [76]. Carboxymethylation is commonly used to improve the water solubility and biological properties of polysaccharides, especially for polysaccharides with poor intrinsic solubility. Accordingly, carboxymethylated derivatives of Poria cocos and Ganoderma lucidum polysaccharides exhibit stronger activity against inflammatory mediators than do the natural polysaccharides themselves [77,78]. Sulfation increases the charge negative density of polysaccharides and can enhance the anti-inflammatory activity of polysaccharides in a degree-of-substitution manner by enhancing interactions with immune-related proteins and receptors [79]. Despite these exciting results, the biological effects of a chemical modification are invariably strongly dependent on the original polysaccharide structure, the modification strategy and the substitution degree, underlining a need for optimisation and systematic development.

Overall, the anti-inflammatory activities of the polysaccharide are the coordinated result of several structural features such as molecular weight, type of monosaccharide units, glycosidic linkage structure, branching structure, higher conformations, chemical modifications, and so on. These structural features differ greatly between various kinds of polysaccharides, accounting for the unique receptor-recognition structures, degradation structure features, pharmacology features, pharmacodynamic mechanisms of different polysaccharides. Thus, general structure–activity relationships can be found, but the biological activity and mechanism of action of individual polysaccharides can only be understood in their own structure feature, not generalized to all kinds of polysaccharide groups. At the same time, current understanding of structure–activity relationships is limited by the structural heterogeneity, divergent extraction and purification methods, inconsistent characterization methods, experimental models etc. of polysaccharides. So these limitations render direct comparisons of these studies difficult and continue to preclude the establishment of universally applicable structure-activity principles, highlighting the need for more standardized analyses and systematic mechanistic studies.

## 4. Anti-Inflammatory Mechanisms of Polysaccharides

Inflammation is a highly coordinated and dynamic biological process triggered by immune-cell activation, by cytokine network amplification, by intracellular signalling amplification and by progressive tissue damage [80]. Growing evidence indicates that the anti-inflammatory action of polysaccharides takes place by various but related mechanisms rather than being centred around one molecule or signalling pathway. Indeed, the mechanisms vary widely among different classes of polysaccharide, and they are closely associated with their structure, receptor-recognition profiles and physicochemical properties.

**Figure 2:**
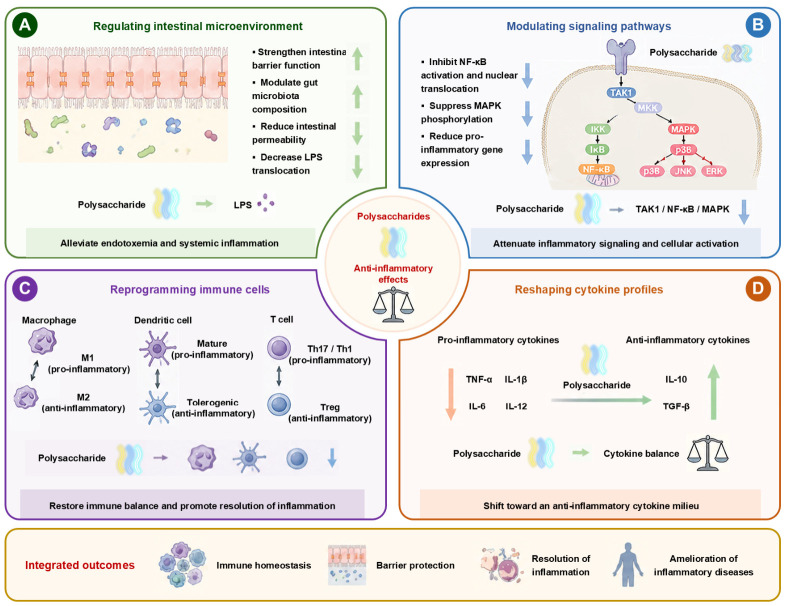
Polysaccharides exert anti-inflammatory effects by reprogramming immune cells, reshaping cytokines, modulating signaling pathways, and regulating the intestinal microenvironment.

Depending on their molecular structures, polysaccharides can directly target immune-cell receptors, can indirectly control inflammation by modulating the gut microbiota and its metabolites, or can be active compounds of drug-delivery systems. Although these different mechanisms can begin in various ways, they all ultimately converge on a few biological phenomena, such as reprogramming immune cells, cytokine network remodelling, intracellular signalling modulation and re-equilibration of the inflammatory microenvironment (Figure 2).

### 4.1. Reprogram Immune Cell Functional States

Immune cells are key regulators of the induction, amplification and resolution of inflammatory responses [81]. They include, for example, macrophages, which are especially important because of their remarkable plasticity in terms of phenotype and can be divided into pro-inflammatory M1 and anti-inflammatory M2. Increasing evidence indicates that the structure of polysaccharides, especially molecular weight, charge and receptor-binding capacity, significantly affects macrophage polarization [82]. Apart from macrophages, polysac carbohydrates also regulate the adaptive immune response. In chronic inflammatory diseases such as atopic dermatitis and inflammatory bowel disease, some polysaccharides suppress Th2- and Th17-mediated inflammatory responses and also restore the levels and immunosuppressive functions of regulatory T cells (Tregs) [83,84]. In sum, all these actions help in suppressing excessive immune responses and maintenance of long-term immune homeostasis.

### 4.2. Reshaping Cytokines Networks

Cytokines are central mediators of communication among immune cells and the damage to tissue during inflammation [85]. Polysaccharides, unlike single cytokines, generally regulate inflammation by modulating the entire cytokine network [86]. Several reports have shown polysaccharides to reduce the production of pro-inflammatory cytokines, such as TNF-α, IL-1β, IL-6, IL-17 and IFN-γ, but, at the same time, increase anti-inflammatory cytokines, including IL-10 and TGF-β [87,88]. For example, suppression of IL-6 production could block downstream JAK/STAT signalling and interrupt positive feedback amplification of inflammatory response. This network regulation is different from single-cytokine blocking. It is more full-bodied immunomodulatory effect and less easily be compensated and enriched for another inflammatory pathway.

### 4.3. Signaling Pathway Modulation

Polysaccharides have the capability to regulate multiple intracellular signal transduction pathways related to inflammation such as NF-κB, MAPK, JAK/STAT and Nrf2/HO-1 pathways [89]. Among these, NF-κB and MAPK are two of the earliest activated signal transduction cascades under inflammatory stimulation. The representative polysaccharides such as hyaluronic acid, fucoidan, β-glucans, Astragalus polysaccharides, have exhibited the suppression of these signal transduction pathways by inhibiting the degradation of IκBα, preventing p65 translocation into nucleus or attenuating the phosphorylation of ERK, JNK, p38, respectively [90,91]. Other polysaccharides have been reported to inhibit JAK2 and STAT3 phosphorylation and promote the expression of suppressor of cytokine signalling 1 (SOCS1) [92,93]. They play a parallel role with antioxidant pathways in inflammation resolution. For example, hyaluronic acid activates the Nrf2/HO-1 signalling pathway to remove ROS in cells, inhibits ROS dependent inflammatory signal pathways and protect tissues from oxidative injuries [94].

Underlying these signals are signals initiated by recognition of polysaccharides by cell-surface receptors. Because of the different structural properties of polysaccharides, different polysaccharides present on cell surfaces are targeted to distinct receptor systems. For example, hyaluronic acid mainly binds CD44 and RHAMM, β-glucans mainly bind Dectin-1, fucoidan mainly binds selectins and scavenger receptors due to its high sulfation level, and several plant- and microbial-sourced polysaccharides have been reported to modulate Toll-like receptors, especially TLR2 and TLR4. Receptor-mediated interactions subsequently orchestrate intracellular signal-activated pathways that ultimately modulate immune-cell activation, cytokine release, oxidative stress and resolution of inflammation. But we should be cautious about interpreting this mechanistic evidence. Although we see modulation of NF-κB, MAPK, JAK/STAT, and Nrf2/HO-1 signalling frequently reported, the key molecular events responsible for triggering these responses are still not fully appreciated. Most reports of mechanistic effects of polysaccharides inferred on the basis of alteration of downstream protein expression or phosphorylation and are not in fact demonstrated directly for receptor binding or target engagement. Besides, some reported signalling changes could well be secondary immune-related consequences rather than direct effect of polysaccharides. For this reason, many proposed mechanisms should be treated as pathway associations rather than mechanisms at present.

### 4.4. Regulating Microenvironment

Polysaccharides have, in addition to regulating immune cells directly, anti-inflammatory actions through modulating the inflammatory microenvironment. One is the remodeling of gut microbiota. For example, HA increases the abundance of beneficial bacterial genera, including Bifidobacterium and Lactobacillus, and reduces pathogens [95,96]. Moreover, many polysaccharides are also substrates for fermentative metabolism by intestinal microbes and promote the production of short chain fatty acids such as acetate, propionate and butyrate, which are important regulators of immune homeostasis and metabolic function [97]. Polysaccharides also strengthen the integrity of intestinal barrier by increasing mucus production and upregulating tight-junction proteins, including ZO-1, occludin, claudin-1 [98]. Strengthening epithelial barrier function decreases intestinal permeability and endotoxin translocation, limiting systemic inflammation and maintaining long-term immune homeostasis.

Overall, polysaccharides regulate inflammation by several complementary mechanisms of immune-cell reprogramming, cytokine network reshaping, signalling circuit-shaping and establishment of an inflammation-modified microenvironment. But important to note is that these mechanisms are not broadly observed for all polysaccharides but depend on structure and are selected depending on receptor recognition, downstream signalling and biological effects. However, considerable progress has been made in uncovering signalling pathways involved, direct molecular targets remain ill defined and most of the mechanistic evidence comes from in vitro experiments and animal models. Future studies of glycomics, structural biology, receptor-identification experiments and multi-omics tools will be essential to decipher the molecular mechanisms of polysaccharide-mediated immune regulation and to pave the way to the rational design of polysaccharide-based anti-inflammatory drugs.

## 5. Polysaccharide-Based Anti-Inflammatory Therapeutic Strategies

### 5.1. Rheumatoid Arthritis

**Figure 3:**
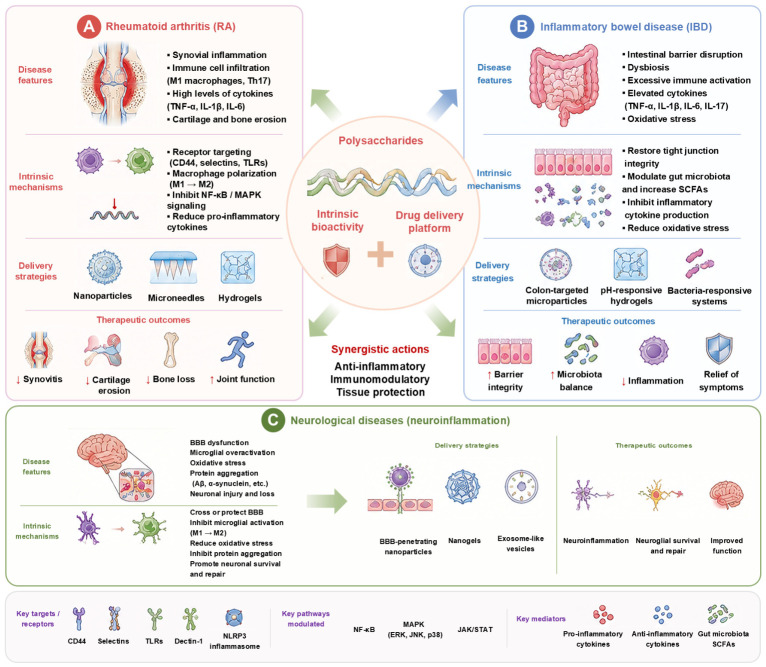
Polysaccharide-based therapeutic strategies for inflammatory diseases. Polysaccharides are used not only as endogenous anti-inflammation agents but also as multifunctional drug-delivery platforms. By receptor-mediated immune modulation, inflammation-responsive signalling, regulation of tissue repair, and targeted drug delivery strategies, polysaccharide-based strategies improve treatment for rheumatoid arthritis, inflammatory bowel disease and neurological diseases. Representative disease-specific mechanisms, delivering strategies and therapeutic targets are highlighted. Figure is original; no copyright permission was required.

Joint-related changes in Rheumatoid arthritis (RA) include chronic synovial inflammation, progressive cartilage degradation, bone erosion, eventually leading to irreversible joint deterioration [99,100]. Although current strategies of treating RA with nonsteroidal anti-inflammatory drugs, glucocorticoids and biologic treatments have significantly improved RA treatment [101], their long-term application can be limited by systemic toxicity, insufficient disease remission, high cost of therapy and side effects [100]. Benefiting from the intrinsic immunomodulatory characteristics, excellent biocompatibility and versatility as biomaterials, polysaccharides could become new candidates for both direct anti-inflammatory strategies and novel drug-delivery therapies for RA (Figure 3).

#### 5.1.1. Polysaccharide-Based Anti-Inflammatory Effects

Polysaccharides effectively alleviate RA and display cooperative regulation at tissue, cell and molecule levels [102]. At the tissue level, polysaccharides coordinately promote the restoration of joint homeostasis and protection of cartilage from chronic progression. For example, hyaluronic acid binds to receptors, such as CD44, to promote lubrication of joints, extracellular matrix stabilization and clearance of inflammatory debris within the extra-cellular space [103]. At the cell level, polysaccharides, such as hyaluronic acid, chitosan and fucoidan, balance macrophage polarization by switching off the pro-inflammatory M1 phenotype and promote the anti-inflammatory and tissue repaired M2 phenotype [104,105]. At the molecule level, polysaccharides attenuate synovial inflammation and hyperplasia via target signalling pathways including NF-κB, MAPK and Nrf2, thus to limit the production of inflammatory mediators and the cartilage damage caused by oxidative stress [106,107].

#### 5.1.2. Polysaccharide-Based Delivery Strategy

Unlike their intrinsic biological activities, polysaccharides have also been extensively engineered into multifunctional drug-delivery materials. In these systems, polysaccharides may not be the actual drug but rather targeting agents, protective matrices, stimuli responsive drug-carrying nanostructures or building blocks to enhance drug stability and tissue retention, sustained drug release and/or drug delivery to a specific tissue or cell. Thus, the therapeutic effect of such systems comes from synergies in both polysaccharide platform and drug cargo.

Among all these materials, HA is most commonly used in the delivery of RA drugs because of its natural abundance in synovial fluid and its affinity for CD44, which is highly expressed in activated macrophages and synovial fibroblasts. Zhu et al. synthesized virus-like nanoparticles consisting of a cyclosporine A loaded nanogel core surrounded by a shell of HA [108]. The HA shell affords CD44-mediated targeting, and the gradual depolymerization in the inflamed joints led to the intracellular release of cyclosporine A and enhanced its therapeutic effect. Likewise, HA-based delivery systems have been developed for targeted delivery of rhein [109], C5 antisense oligonucleotide [110] and mRNA [111]. Here, HA acts mainly as the targeting moiety and as a biocompatible carrier capable of augmenting intra-articular retention and cellular uptake, but the major drug functions stem from the encapsulated therapeutic materials.

Chitosan is another widely investigated polysaccharide for RA drug delivery because of its cationic properties, mucoadhesive properties and facile chemical modification. Chitosan-stabilized nanoparticles significantly ameliorated inflammatory cell infiltration, synovial hyperplasia and cartilage degradation in collagen-induced arthritis mouse model [112]. Apart from that, chitosan has also been incorporated into transdermal microneedles for continuous monitoring of the methotrexate concentration [113] and in a pH-responsive composite hydrogel for delivery of melittin to treat inflamed joints [114], showing the versatility of chitosan in both therapy and monitoring purposes.

Dextran-based systems represent another example of structurally tunable drug-delivery systems [115]. Following their chemical tunability, dextran derivatives have been developed as pH-responsive, redox-responsive and macrophage-targeted delivery carriers capable of selectively delivering therapeutics to inflamed joints [115,116]. For instance, sulfated dextran-modified nanoparticles for delivery of the therapeutic S-nitrosoglycine showed enhanced macrophage accumulation and effectively reduced intra-cellular ROS concentrations, suppressed NF-κB induction, inhibited the M1 activation of macrophages and reduced the production of TNF-α [117]. Besides these representative examples, some other polysaccharides (such as fucoidan) have also been combined with targeted nanoparticles, injectable hydrogels and microneedle systems for local delivery of treatment for RA. These works demonstrate the versatility of polysaccharides as multifunctional biomaterials with abilities to bring disease targeting, programmed drug release and intrinsic immunoregulation ability.

Despite encouraging results for some of these promising, polysaccharide-based therapeutic approaches to RA, most polysaccharide therapeutic strategies are currently at a preclinical level. Comparisons between studies are further complicated by a large variability in polysaccharide source, characteristics, formulation, animal models, dosing and outcome measurements. Moreover, despite several hyaluronic acid formulations entering clinical trials for osteoarthritis, clinically compelling evidence for polysaccharide-based therapeutic delivery for RA is limited. Future clinical translation will require standardised characterisation of polysaccharides, appropriate comparative studies, and well-powered clinical trials to demonstrate efficacy and safety over the long term.

### 5.2. Inflammatory Bowel Disease

Many diseases, such as inflammatory bowel disease (IBD), are chronic and relapsing gastrointestinal diseases with persistent mucosal inflammation, damaged epithelium barrier, immune disorder and dysbacteriosis in gut microbiota [118]. It has made considerable progress in therapies such as aminosalicylates, corticosteroids, immunosuppressants and biologics, but the cure of current IBD treatment is limited by incomplete remission, frequent relapse, side effects and high drug costs [119]. Polysaccharides have the favourable characteristics of excellent biocompatibility, oral delivery, inherent immunoregulatory properties and interaction with the intestine microenvironment, and thus are promising candidates for both the direct application in treating IBD and colon-targeted drug-delivery system (Figure 3).

#### 5.2.1. Polysaccharide Exert Therapeutic Effects

Restoration of intestinal barrier: Disruption of intestinal epithelial barrier is a major pathological mechanism of IBD progression. Polysaccharides protect intestinal epithelial barrier integrity by upregulating intestinal tight-junction proteins, such as ZO-1, occludin and claudin-1, inhibiting the apoptosis of epithelial cells and increasing mucin secretion [120,121]. For example, hyaluronic acid rectal application protected intestinal mucosa from experimental colitis by suppression of inflammation, declined intestinal epithelial permeability and maintenance of intestinal barrier integrity [122]. Polysaccharides extracted from *Atractylodes macrocephala* also upregulated the expression of intestinal tight-junction proteins and improved intestinal mucosal repair in experimental IBD [123].

Suppression of inflammatory responses. Many polysaccharides can relieve intestinal inflammatory reactions by suppressing relevant inflammatory pathways such as TLR4/MyD88/NF-κB, MAPK and NLRP3 inflammasome. Polysaccharides extracted from Lentinula edodes, *Astragalus membranaceus* and Dendrobium officinale could substantially reduce experimental colitis by reducing NF-κB activation [124]. Similarly, inhibition of NLRP3 inflammasome activation reduced the secretion of pro-inflammatory factors, IL-1β and IL-18, and therefore mitigate epithelial damage and mucosal inflammation [125]. Furthermore, chemical modification can also further enhance therapeutic function. For example, sulfation can enhance the antioxidant activity and free radical scavenging activity of Sargassum pallidum polysaccharides and make its anti-inflammatory effect much better [126].

Regulation of immune function: Immune dysregulation, especially the imbalance of effector T-cell subset and regulatory T cells (Tregs), contributes to the pathogenesis of IBD [127]. Thus, restoration of T-cell homeostasis is an important intervention strategy. Astragalus polysaccharide inhibited Th17 cell differentiation and stimulated Treg proliferation, reduced inflammatory cytokine release and alleviated experimental colitis by restoring T cell homeostasis [128]. Likewise, polysaccharides extracted from Ganoderma lucidum [129] and *Atractylodes macrocephala* [130] could alleviate intestinal inflammation by balancing Th1/Th2 and Th17/Treg cell homeostasis, contributing to persistent inhibition of intestinal inflammation and regulation of immune homeostasis.

Modulation of gut microbiota: Gut microbiota dysbiosis, characterised by reduced microbiota diversity and bacterial SCFA-production depletion, is a feature of IBD [131]. As natural fermentation substrates, polysaccharides shape the intestinal microbiota and modulate microbial metabolism. Chitosan, alginate, hyaluronic acid and some plant polysaccharides have been reported to enhance the beneficial bacteria, including Lactobacillus, Bifidobacterium, Lachnospiraceae and Ruminococcaceae, whilst decreasing the possible harmful bacteria such as Escherichia-Shigella, Proteobacteria and Bacteroides [132,133,134,135]. These changes in gut microbiota give rise to an increase in SCFAs, repair intestinal epithelial energy metabolism, strengthen epithelial barrier integrity and alleviate the chronic inflammatory response.

#### 5.2.2. Polysaccharide-Based Delivery Strategy

Similarly to rheumatoid arthritis, polysaccharides have been widely developed as multifunctional materials for colon-targeted drug delivery. Instead of being therapeutic drugs themselves, they are mostly serving as protective materials, pH-sensitive materials, microbiota-responsive materials or target materials enhancing the gastrointestinal stability, colon-targeted drug release and local drug retention of drugs. The therapeutic efficacy of such delivery systems thus mainly come from the additive effects of the polysaccharide matrix and the encapsulated therapeutic drug, respectively.

Alginate is one of the most widely studied colon-targeting materials because it can form pH-sensitive hydrogels by ionic crosslinking with divalent cations such as Ca^2+^ [136,137]. The hydrogels are stable in the gastric environment, but gradually swell or degrade in the intestine and thus enable colon-specific drug release. For example, calcium alginate hydrogels for hyaluronic acid-modified selenium nanoparticles enabled in vivo selenoprotein synthesis, regulation of pro-inflammatory cytokines and mitigating experimental colitis [138]. Alginate has been used for delivery of Cas9/sgRNA ribonucleoproteins [139] and probiotics [140]. In these applications, alginate merely provides protection and pH responsiveness as a carrier, while the substantial activity is due to the biological function of the cargos loaded.

Stimulus-responsive polysaccharides have even further expanded the applicability of colon-specific drug-delivery systems. For example, ROS-sensitive micelles prepared from hyaluronic acid–stearic acid conjugates selectively released the STING inhibitor, RU.521, from inflamed intestinal tissue after ROS-induced cleavage of the thioketal linkers [141]. Similarly, thiolated hyaluronic acid cross-linked into an ROS-sensitive hydrogel-coated layer for the inflamed intestinal mucosa in response to oral intake, thus reducing microbial invasion and excessive immune activation [76]. In addition, dopamine-modified hyaluronic acid has also been developed for IBD treatment [76].

Also, because polysaccharides are degradable by gut microorganisms, microbiota-responsive drug delivery systems can be realized. Ma et al. engineered a GlycoCaging platform by coupling plant glycoconjugates to anti-inflammatory drugs, which can release the drugs in the colon by the microbes-induced glycosidases in microbiota [136]. Similarly, an inulin hydrogel loaded with polypyrrole nanozyme and pirfenidone was degraded selectively by the microbiota in the colon, which gave the drug-release effect on site, suppressed inflammatory cytokine production, recovered epithelial barrier function and suppressed intestinal fibrosis both in acute and chronic colitis models [120].

Despite these promising preclinical findings, most polysaccharide-based therapies for IBD remain experimental. Direct comparison between studies is challenging because of substantial differences between polysaccharide source and structure, microbiota composition, disease models, administration type and method of evaluation. In addition, because gut microbiota composition differs substantially between rodent and human hosts, the therapeutic efficacy demonstrated in experimental models of colitis will not necessarily reflect clinical outcomes. Although some polysaccharide formulations have progressed into initial clinical evaluation, strong evidence for large-scale randomized clinical trials is still sparse. Future translation will thus require thorough polysaccharide characterization, harmonization of preclinical models and well-designed clinical studies to assess efficacy, safety and long-term therapeutic benefit.

### 5.3. Neurological Diseases

Neurodegenerative diseases, including Alzheimer’s disease, Parkinson’s disease and Huntington’s disease, are progressive, incurable disorders featuring gradual death of neurons in structure and function [142]. Neurodegenerative diseases result in cognitive disorders, motor function disorder and behavioural disorder, causing huge troubles for the patients, families and health-care systems. Although great research efforts have been made, available therapies have still only palliative roles and limited ability to counter or reverse the progresses of neurodegenerative diseases. Because of their structural diversity, good biocompatibility and various biological functions, natural polysaccharides are of increasing importance as potential therapeutic agents as well as favourable drug-delivery materials for treating neurodegenerative diseases (Figure 3).

#### 5.3.1. Neuroprotective Activities of Polysaccharides

Polysaccharides can modulate a number of pathological processes related to neurodegenerative diseases. Sulfated and acidic polysaccharides (alginate, fucoidan and glycosaminoglycans) have been shown to inhibit the aggregation of amyloid-β and α-synuclein, two characteristic pathogenic proteins of Alzheimer’s disease and Parkinson’s disease, respectively [143,144,145]. Additionally, several of the naturally derived polysaccharides are acetylcholinesterase and butyrylcholinesterase inhibitors that increase acetylcholine levels and can improve memory and cognitive abilities. Beyond the protein aggregation, oxidative stress and neuroinflammation are another class of major therapeutic targets. Polysaccharides isolated from seaweed, fungi and medicinal plants have been reported to scavenge ROS, increase endogenous antioxidant systems, such as superoxide dismutase, glutathione peroxidase and glutathione, and maintain mitochondrial membrane potential [143,146]. Moreover, many polysaccharides were shown to suppress microglial activation and to downregulate inflammatory pathways, such as NF-κB, MAPK and TLR4/MyD88 signalling, to promote an anti-inflammatory microglial state [147,148].

Gut–brain axis is another mechanism of the neuroprotective action of polysaccharides. By modulating the composition of gut microbiota, increasing the production of short-chain fatty acids, repairing the intestinal barrier and attenuating peripheral inflammation, polysaccharides can indirectly modulate central nervous system homeostasis. For example, Eucommiae cortex polysaccharides could improve neurological outcome by improving gut microbiota composition and intestinal barrier function [149], ulva polysaccharides could alleviate Parkinson’s disease progression by modulating gut microbiota [150]. These results show that altering gut-brain axis might be a unique pharmacological advantage of polysaccharides compared with existing neuroactive drugs.

#### 5.3.2. Polysaccharide-Based Delivery Strategy for Neurological Diseases

Apiece with the intrinsically neuroprotective activities of polysaccharides, this class of polymers has received much interest as multifunctional delivery materials for CNS disorders13. For these systems, the polysaccharides solely improve the drug delivery ability by enhancing biocompatibility, BBB-penetration ability, stability of the carrier and initiating stimulus-responsive drug release and, frequently, the main pharmacology is conferred by the cargo drugs themselves.

As BBB is one of the major barriers of drug delivery to the central nervous system [151], many chemically modified polysaccharides have been developed to promote BBB transport or facilitate targeting to disease sites. For example, Wang and co-workers engineered a sulfated polysaccharide nanogel that could penetrate the BBB and release therapeutic cargos in a targeted way due to their high levels of ROS in diseased sites [152]. Similarly, fucoidan-derived carbon dots can pass the BBB very efficiently and alleviate PC12 cell injury by virtue of their combined anti-inflammatory, antioxidant and anti-apoptotic actions [153]. In these nanoplatforms, polysaccharides mainly participate in BBB-transporting, carriers’ stableness and targeting efficiency, and the overall effects are in general from joint contributions of delivery platforms and loaded therapeutic cargos.

However, despite these encouraging results, evidence for polysaccharide-based therapies for neurodegenerative diseases that is available today remains essentially preclinical. Most mechanistic studies have been done in cultured cells and animal models, and a few have explored long-term efficacy, pharmacokinetic properties, BBB transport efficiency or safety in a clinically relevant setting. Furthermore, the very complicated pathogenesis of neurodegenerative diseases and substantial differences that exist between experimental models of neurodegenerative diseases and patients mean that direct translation of preclinical results is not readily achievable. Robust clinical evidence to support successful therapy is therefore scarce and further translational studies and carefully designed clinical trials are needed before polysaccharide-based therapies can realistically be proposed as clinical entities.

Overall, however, the therapeutic roles of polysaccharides are different for the different intrinsic biological properties and physicochemical characteristics of each polysaccharide. Some polysaccharides are therapeutically active in the form of bioactive agents, and other polysaccharides can serve more as targeting ligands, drug carriers, protective templates or stimulus-sensitive biomaterials. In this context, the therapeutic effect of a polysaccharide-based system should be interpreted according to the specific role played by the polysaccharide in each of these systems, rather than being entirely linked to the polysaccharide. Further studies should differentiate between the respective roles of the polysaccharide and encapsulated therapeutic agents, strengthen mechanistic proof-of-concept and clinical translation to rationalize the development of polysaccharide-based therapeutics for neurological diseases.

## 6. Current Challenges and Future Perspectives

Although major advances have been made in unravelling the anti-inflammatory activities of polysaccharides, the overall quality and homogeneity of the available evidence still remain highly heterogeneous and continue to hamper clinical translation. Differences in polysaccharide source, extraction and purification process, structural characterisation, experimental models, outcome measures, reporting, etc. make comparison among studies difficult and impede reproducibility. In addition, positive results have been reported far more often than negative or inconclusive results and introduce a possible publication bias and likelihood of reporting overestimated treatment effects. Together with structural heterogeneity, lack of pharmacokinetic knowledge, insufficient validation of mechanisms of action, low or non-standard quality-control standards, as well as paucity of high-quality clinical evidence, these concerns present important challenges to the development of polysaccharide therapeutics (Table 2). Resolving these issues will require consistent structural characterisation, well-controlled quality standards, well-designed mechanistic studies with direct validation of the target, as well as well-designed translation-oriented clinical studies.

### 6.1. Current Clinical Evidence and Translational Status

Despite the encouraging preclinical evidence that we review here, relatively few polysaccharide-based anti-inflammatory therapies have reached the clinic so far. Currently, convincing clinical evidence exists for only a few naturally occurring polysaccharides, while most new polysaccharide formulations and delivery technologies are at the preclinical level of development. Among the currently available polysaccharides, HA has enjoyed the highest level of clinical translation. Currently, intra-articular injection of HA is widely used to treat OA due to its viscoelasticity, lubrication property, and local immunomodulatory effect. Besides the conventional viscosupplementation approach, a few HA-based polymer–drug conjugates such as diclofenac–etalhyaluronate and HA–plasma-rich fibrin formulations also showed prolonged intra-articular retention and enhanced therapeutics with their efficacy, and several formulations are undergoing clinical trials in Phase II or III. These advances both highlight the dual role of HA as a bioactive therapeutic molecule and as a highly versatile drug-delivery platform.

Chondroitin sulfate also has rather robust clinical evidence, particularly for osteoarthritis, where multiple randomised clinical trials have already demonstrated improvements in pain relief and joint function [154]. But the level of clinical benefit is still controversial because of huge variations in product sources, molecular forms, manufacturing quality, dosage, treatment duration and study designs. Thus, although HA and CS products have come into clinical practise for selected musculoskeletal diseases, their efficacy is still under debatable practice. On the contrary, most of the other polysaccharides (including fucoidan, alginate, chitosan, β-glucans, Astragalus polysaccharides, pectin and konjac glucomannan) are proven mainly from in vitro and animal studies. Similarly, most advanced polysaccharide-based formulations—including polysaccharide-based nanoparticles, injectable hydrogels, microneedles and stimulus-responsive delivery systems—show promising therapeutic results in preclinical studies but have rarely advanced to clinical studies.

Overall, current clinical evidence supports the translational potential of polysaccharides, but it is insufficient to support the widespread adoption of clinical recommendations for most inflammatory diseases. Progress in the future will rely on multicentre randomised controlled trials including standardised polysaccharide characterisation, harmonisation of polysaccharide manufacturing procedures, pharmacokinetic testing, assessment of long-term safety and clinically relevant trial end points.

### 6.2. Challenges in Polysaccharide-Based Anti-Inflammatory Strategy

#### 6.2.1. Structural Complexity

Polysaccharides are natural heterogeneous macromolecules that can vary in molecular weight, monosaccharide composition, glycosidic linkage type, type of branching architecture and chemical modifications [155]. Because polysaccharide biosynthesis is not template-directed, minor differences in biological source or extraction procedure can strongly influence structure and/or biological activity. Accordingly, limited analytical resolution and widespread employment of poorly characterized polysaccharide fractions can obscure structure–activity relationship, lack reproducibility and hamper rational therapeutic design [156].

#### 6.2.2. Limited Understanding of In Vivo Fate

For most polysaccharides, pharmacokinetic properties are poorly understood. Basic questions of absorption, biodistribution, metabolism, tissue retention and clearance remain largely unanswered and an appropriate methodology to assess the PK/PD characteristics of structurally diverse polysaccharides is still missing. Without a firm grasp of exposures, any optimization of doses, any prediction of efficacy and translation into the clinic will remain difficult.

#### 6.2.3. The Double-Edged Sword of Multi-Target

Polysaccharides have a multi-target nature, which is an important therapeutic advantage and a major mechanistic drawback [157]. Although processes like NF-κB, MAPK, JAK/STAT, TLR signalling have often been implicated [158], direct molecular targets have not been verified experimentally very often. Moreover, it is often difficult to understand if observed biological effects are due to direct interactions with a receptor, secondary immune regulation or even just metabolites of the microbiota. Hence, many reported mechanisms should currently be understood as pathway associations, rather than mechanistic causalities.

**Table 2:** Structural heterogeneity, characterization, and standardization challenges of polysaccharides.

Aspect	Key Factors	Challenges	Impact on Research and Translation	Potential Solutions	Reference
Source variability	Species, geographical origin, cultivation conditions, harvest season	Inconsistent composition and bioactivity	Poor reproducibility across studies	Controlled sourcing; standardized raw material selection; documentation of species, origin, cultivation conditions, and harvest season	[159,160]
Extraction and purification	Solvent type, temperature, pH, enzymatic treatment, purification strategy	Altered molecular weight, branching architecture, and functional groups	Difficulty in comparing results between studies	Standardized extraction and purification protocols; detailed reporting of extraction conditions, purification procedures, and extraction yields	[161,162]
Structural heterogeneity	Monosaccharide composition, glycosidic linkages, branching patterns, molecular weight, sulfation degree	Lack of precise structural definition	Unclear structure–activity relationships	Advanced synthesis and fractionation strategies; comprehensive structural characterization of molecular weight, monosaccharide composition, glycosidic linkages, branching architecture, and sulfation degree	[163,164]
Characterization limitations	Limited analytical resolution and incomplete structural characterization	Incomplete structural information	Limited mechanistic understanding	Integrated analytical platforms combining NMR, mass spectrometry, chromatography, and glycomics to improve structural characterization	[164,165]
Batch-to-batch variability	Raw material variability and manufacturing processes	Inconsistent quality and biological performance	Barriers to clinical development and industrial production	Process control and batch consistency assessment based on predefined critical quality attributes (e.g., molecular weight distribution, monosaccharide composition, sulfation degree, and bioactivity)	[164,165]
Quality control	Lack of defined critical quality attributes	Difficulty in establishing release standards	Regulatory uncertainty and inconsistent product quality	Establish critical quality attributes, including molecular weight distribution, monosaccharide composition, sulfation degree, endotoxin levels, residual protein/nucleic acid content, sterility, stability, and bioactivity assays	[166,167]
Standardization	Absence of unified analytical and reporting guidelines	Poor comparability across studies	Hinders regulatory approval and clinical translation	Development of reference standards, standardized reporting guidelines, and consensus analytical methods	[168,169]
Regulatory considerations	Classification ambiguity (drug, biologic, natural product, excipient, or drug-delivery material)	Unclear approval pathways	Delayed clinical translation	Early alignment with GLP/GMP requirements and regulatory frameworks according to the intended therapeutic application	[170]

#### 6.2.4. Translational Barriers from Preclinical Models to Clinical Application

Most polysaccharide-based anti-inflammatory methods have been demonstrated in vitro studies or rodent models, which are far removed from human diseases in terms of immune responses, microbiota composition and disease development [171]. Large-scale production, formulation stability, manufacturing quality control and registration issues still remain. Depending on the actual use, polysaccharides may be regulated as active pharmaceutical ingredients, biologics, excipient or drug delivery material and correspondingly follow different regulatory ways and have distinct quality requirements.

#### 6.2.5. Source Variability and Standardization Challenges

Source variability also adds to the complexity of polysaccharide development. Species, geographical origin, cultivation condition, harvest time, extraction procedures, purifying techniques, storage conditions can all be variables affecting composition and biological activity, giving large differences between samples of the same product (‘batch effects’). Contaminants such as endotoxins, residual proteins, residual nucleic acids, impurities introduced during purification all can have an effect on product efficacy and safety. Thus critical quality attributes, validation of analytically standardized methods and consensus quality-control and quality-assurance standards will have to be defined for reproducibility, regulatory purposes and industry application.

### 6.3. Future Directions

#### 6.3.1. Precision Glycoscience and Structural Engineering

Future progress will also depend on greater control over polysaccharide structure. Chemoenzymatic synthesis, glycosyltransferase engineering and advanced glycoengineering strategies will provide structurally defined polysaccharides, and combined integrated analytical approaches, using NMR spectroscopy, mass spectrometry, structural biology and computational modeling, can enable more reliable structure–activity analyses.

#### 6.3.2. Intelligent Delivery Systems

Future delivery platforms should incorporate an intrinsic polysaccharide bioactivity in combination with a disease-responsive drug delivery platform. Smart systems that respond to pathological signals, such as an acidic pH, reactive oxygen species, matrix metalloproteinase or inflammatory enzymes, will drive a site-specific drug release with low system toxicity.

#### 6.3.3. Deepening Mechanistic Studies

Future mechanistic studies should move beyond describing downstream events of signalling and instead concentrate on defining direct molecular targets and receptor-recognition mechanisms. Having a better understanding of how molecular weight, the relative content of monosaccharide residues, glycosidic linkages, branch structures, sulfation, and other architectural features of glycans define the receptor-binding properties and signal induction is likely to greatly reinforce structure–activity relationships. Combining receptor-binding assays, structure biology, glycomics, single-cell transcriptomics and -proteomics, metabolomics and AI-driven molecular modelling can give us a more holistic view of polysaccharide-mediated immune regulation.

#### 6.3.4. Reframing Research Paradigms

Future preclinical studies should use disease models that more closely mimic human inflammatory diseases, should include a robust structural characterization, pharmacokinetics, assessment of immunogenicity and safety or toxicity tests, align early with regulatory guidelines and good manufacturing practice guidelines, to facilitate subsequent clinical development.

#### 6.3.5. Interdisciplinary Integration

Ultimately, successful clinical translation will require closer collaboration between glycoscience, structural biology, immunology, computational biology, synthetic biology, materials science, pharmaceutical engineering, clinical medicine, and others. The interdisciplinary blending described above can speed up standardizing manufacturing, mechanistic discovery, regulatory clearance and moving on to the next-generation polysaccharide-based anti-inflammatory drugs.

## 7. Conclusions

Polysaccharides are a class of biopolymers with diverse structures and promise for the treatment of inflammatory diseases. Acting not only as intrinsic anti-inflammatory agents but also as multifunctional biomaterials for drug delivery, they mediate inflammation via concurrent modulation of immune-cell function, cytokine network, intracellular signalling pathways, epithelium and tissue barriers and the gut microbiota. However, their biological activities are structure-dependent and have to be assessed in the context of each individual polysaccharide rather than analogously to all polysaccharide classes. Although great progress has been made, clinical translation remains limited owing to structural heterogeneity, insufficient mechanistic validation, insufficient understanding of the pharmacokinetics and the lack of solid characterization and high-quality clinical evidence. Greater advances in precision glycoscience, structural characterization, intelligent delivery vehicles and translation-oriented research will be needed to close the gap between promising preclinical evidences and clinical application. Interdisciplinary working, polysaccharide-based therapeutics have great potential to become a new generation of safe, multifunctional, and clinically useful anti-inflammatory therapies.

## Data Availability

No data was used for the research described in the article.
